# COVID-19 Vaccines during Pregnancy and Breastfeeding: A Systematic Review

**DOI:** 10.3390/jpm13010040

**Published:** 2022-12-25

**Authors:** Blanca Novillo, Alicia Martínez-Varea

**Affiliations:** Department of Obstetrics and Gynaecology, La Fe University and Polytechnic Hospital, Avenida Fernando Abril Martorell 106, 46026 Valencia, Spain

**Keywords:** breastfeeding, pregnancy, COVID-19, vaccines, immunoglobulin

## Abstract

**Background**: Pregnant and breastfeeding women received unclear recommendations regarding COVID-19 vaccination at the beginning of the pandemic, as they were not included in the initial clinical trials. This systematic review aims to provide an update regarding COVID-19 vaccines during pregnancy and breastfeeding. **Methods:** The systematic review was carried out through a literature search in Medline/Pubmed. Studies were selected if they included information regarding COVID-19 vaccination during pregnancy and breastfeeding. The PRISMA guidelines for systematic reviews were followed. **Results:** A total of 33 studies were included. The main adverse effect is pain at the injection site, as in the general population. Adverse effects are more frequent after the second dose, being slightly more frequent after the Moderna vaccine. COVID-19 vaccination reduces the risk of severe COVID-19 in pregnant women. Additionally, COVID-19 vaccination induces artificial active immunogenicity in the mother and natural passive immunogenicity in the child. Breastmilk straddles both immediate antibody-mediated and long-lived cellular-mediated immune protection. Regarding neonatal benefits, vaccination is associated with a larger and more stable Immunoglobulin G response, while COVID-19 Infection is associated with a rapid and long-lasting Immunoglobulin A response. **Conclusions:** COVID-19 vaccines are not only suggested but strongly recommended for pregnant and breastfeeding populations to protect mothers and newborns.

## 1. Introduction

Pregnancy is a stressful situation for the human body. The pregnant woman undergoes well-known physiological changes in her respiratory and cardiovascular systems [1,2]. Pregnancy is associated to a procoagulant and proinflammatory state [3,4]. Moreover, the maternal immune system has to achieve an immune tolerance toward the fetus [3], which entails a decrease in responses to viral infections [3]. All of these features make pregnant women, particularly those with chronic diseases, susceptible to severe COVID-19 disease [5,6]. 

Pregnant women develop a more severe COVID-19 disease compared to non-pregnant patients [7]. In addition, pregnant patients with SARS-CoV-2 infection experience a higher rate of preterm birth, cesarean birth, and stillbirth compared to non-infected pregnant women [8,9]. Moreover, COVID-19 during pregnancy has been associated with preeclampsia [10,11], especially among nulliparous women [11].

The best strategy to fight against infections is immunization through vaccination [7]. This becomes even more efficient in pregnant and lactating women, achieving double immunization with a single administration: active artificial for the mother and passive natural for the fetus [12].

Nonetheless, pregnant and lactating women received unclear recommendations regarding COVID-19 vaccination at the beginning of the pandemic, because they were not included in the first clinical trials. Accordingly, the acceptance of the COVID-19 vaccine in the pregnant and lactating populations has been reported to be limited [13,14,15,16]. This systematic review aims to analyze the growing body of evidence regarding the safety, efficacy, and immunogenicity of SARS-CoV-2 vaccination during pregnancy and lactation. 

## 2. Materials and Methods

This systematic review was carried out according to PRISMA guidelines [17,18]. The search guidelines used were MEDLINE/ PUBMED. The search terms used were “COVID-19 vaccines AND pregnancy” and “COVID-19 vaccines AND breastfeeding”. ZOTERO was used to arrange articles and eliminate duplicates. The literature search was performed, and all published studies from the 1 December 2019 to the 5 December 2022 were initially selected. 

Inclusion criteria were studies regarding COVID-19 vaccines and pregnant and/or breastfeeding women as well as quantitative studies. Articles regarding pregnant women were included regardless of the week of gestation. Studies concerning breastfeeding women were also included, irrespective of the age of the children. The exclusion criteria were review articles, non-human studies, and studies with less than 10 participants. There were no language restrictions. 

A second researcher double-checked that the selected abstracts met the criteria. Disagreements were resolved by discussion and consensus. Data were then collected by reading the articles that were finally included.

## 3. Results

**Figure 1:**
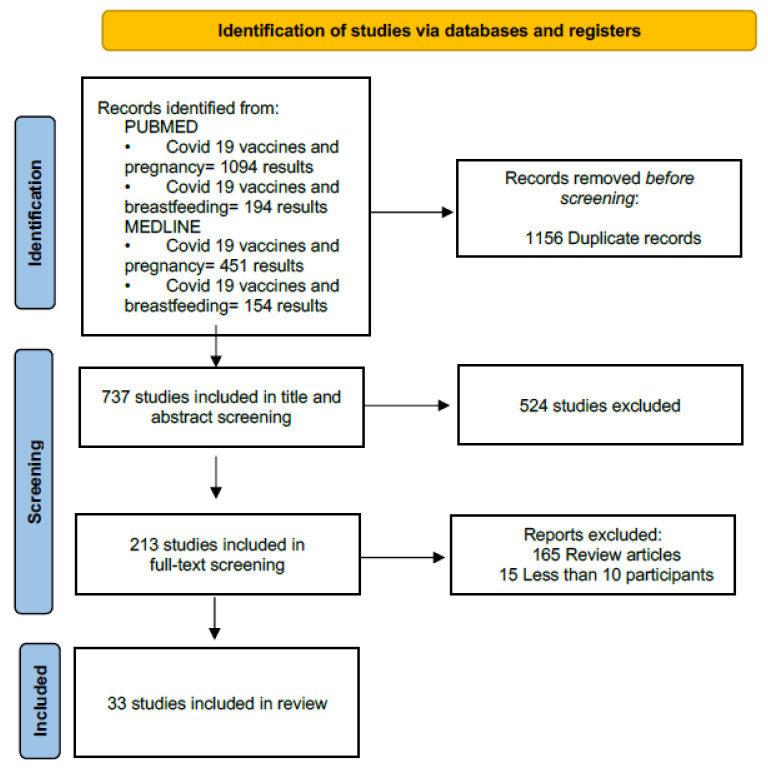
PRISMA: preferred reporting items for systematic reviews and meta-analyses.

Combined database searches yielded 1893 articles, as shown in Figure 1. A total of 33 studies were ultimately included. The PRISMA flow chart shown in Figure 1 reveals the search process for the systematic review.

Study characteristics are described in Table 1. The vast majority of studies had a cohort design. Three studies used a cross-sectional design [19,20,21], and two used a case-control design [22,23].

Regarding the comparison of vaccines, most studies compare vaccines made by messenger ribonucleic acid (mRNA) technology: Pfizer (BNT162b2) and Moderna (mRNA-1273). However, four studies included the AstraZeneca vaccine (ChAdOx1-S) [20,24,25,26], a Brazilian study included the CoronaVac (Biotech) vaccine [27], and one study included the Janssen (Johnson & Johnson) (NCT04505722) vaccine [23].

**Table 1:** Characteristics of the studies included in the systematic review.

Author	Population	N	Study Design	Vaccine Type	No Dose	Country	Date of Publication
Atyeo [28]	Pregnant, breastfeeding and non-pregnant.	131	Observational cohort	Pfizer or Moderna	2	USA	10/2021
Bertrand [29]	Breastfeeding vaccinated	180	Observational cohort	Pfizer or Moderna	1 or 2	USA	08/2021
Calil [27]	Breastfeeding vaccinated	20	Observational cohort	CoronaVac (Biotech)	2	Brazil	06/2021
Charepe [30]	Breastfeeding and not	24	Observational cohort	Pfizer	2	Portugal	09/2021
Collier [31]	Pregnant and breastfeeding vaccinated and unvaccinated infected	131	Observational cohort	Pfizer or Moderna	Does not specify	USA	03/2021
Esteve-Palau [32]	Breastfeeding vaccinated	33	Observational cohort	Pfizer	2	Spain	08/2021
Rosenberg-Friedman [33]	Breastfeeding vaccinated	10	Observational cohort	Pfizer	2	Israel	03/2021
Golan [34]	Breastfeeding vaccinated	50	Observational cohort	Pfizer or Moderna	2	USA	11/2021
Gonçalves [35]	Breastfeeding, vaccinated	23	Observational cohort	Pfizer or Moderna	2	Portugal	12/2021
Gray [36]	Pregnant, breastfeeding and non-pregnant.	131	Observational cohort	Pfizer or Moderna	1 or 2	USA	09/2021
Guida [37]	Breastfeeding, vaccinated	10	Observational cohort	Pfizer	2	Italy	07/2021
Jakuszko [38]	Breastfeeding vaccinated and not vaccinated	60	Observational cohort	Pfizer	2	Poland	06/2021
Juncker [39]	Breastfeeding vaccinated	26	Observational cohort	Pfizer	2	Netherlands	08/2021
Kachikis [40]	Pregnant, breastfeeding and planning pregnancy vaccinated.	17,525	Observational cohort	Pfizer or Moderna	1 or 2	USA	08/2021
Kadali [19]	Pregnant vaccinated	38	Cross-sectional	Pfizer or Moderna	1 or 2	USA	10/2021
Lechosa-Muñiz [20]	Breastfeeding vaccinated	110	Cross-Sectional	Pfizer, Moderna, or AstraZeneca	1 or 2	Spain	08/2021
Low [41]	Breastfeeding, vaccinated or unvaccinated or infected	25	Observational cohort	Pfizer	2	Singapore	08/2021
McLaurin-Jiang [21]	Breastfeeding vaccinated	4455	Cross-sectional	Pfizer or Moderna	1 or 2	USA	06/2021
Mithal [42]	Pregnant vaccinated	27	Observational cohort	Pfizer or Moderna	1 or 2	USA	08/2021
Montalti [43]	Breastfeeding and pregnant	600	Observational cohort	Pfizer	2	Italy	08/2021
Olearo [44]	Breastfeeding, vaccinated or not, having had de infection or not	21	Observational cohort	Pfizer	1 or 2	Germany	09/2022
Perez [45]	Pregnant or breastfeeding vaccinated	30	Observational cohort	Pfizer or Moderna	1	USA	02/2022
Perl [46]	Breastfeeding vaccinated	84	Observational cohort	Pfizer	2	Israel	04/2021
Pietrasanta [47]	Breastfeeding vaccinated and their babies	24	Observational cohort	Pfizer	2	Italy	06/2022
Prabhu [48]	Pregnant vaccinated	122	Observational cohort	Pfizer or Moderna	1 or 2	USA	04/2021
Rottenstreich [49]	Pregnant vaccinated	20	Observational cohort	Pfizer	2	Israel	04/2021
Scrimin [50]	Breastfeeding vaccinated, infected or not	42	Observational cohort	Pfizer, Moderna, or AstraZeneca	1 or 2	Italy	01/2022
Selma-Royo [25]	Breastfeeding vaccinated vs Breastfeeding not vaccinated	86	Observational cohort	Pfizer, Moderna, or AstraZeneca	2	Spain	04/2021
Shanes [22]	Pregnant vaccinated and unvaccinated	200	Case control	Not mentioned	Not mentioned	USA	08/2021
Shimabukuro [51]	Pregnant vaccinated	35,691	Observational cohort	Pfizer or Moderna	1 or 2	USA	04/2021
Theiler [23]	Pregnant vaccinated vs not vaccinated	2002	Case- control	Pfizer, Janssen, or Moderna	1 or 2	USA	11/2021
Young [17]	Breastfeeding vaccinated or infected	77	Observational cohort	Moderna or Pfizer	2	USA	11/2021
Martínez-Varea [26]	Pregnant infected vaccinated or not	487	Observational cohort	Pfizer, Moderna, or AstraZeneca	2	Spain	12/2022

### 3.1. Safety

A total of 9 articles have been found that discussed the side effects of vaccination [19,20,21,22,23,29,40,43,51]. Most of them detected no [22] or minor side effects. In the largest prospective cohort (17,525 patients), the main side effects were pain at the injection site (92%) and fatigue (30% and 70% after the second dose) [40]. These side effects were similar to the general population [19]. Among lactating women, decreased milk supply <24 h was described in 7%, with few or no subsequent repercussions, and 2% presented interrupted breastfeeding [40]. Other side effects were general malaise (18.2% of the women), adenopathy (18.2%), headache (9.1%), fever (6.4%), and nausea (0.9%). No side effects were described in 34.5% of the women (20).

Slightly more adverse effects and decreased milk supply have been reported with the Moderna vaccine [29]. A Spanish study administered the AstraZeneca vaccine to 18.2% of the 110 patients. The most frequent side effects of the AstraZeneca vaccine were general malaise and lymphadenopathy [20]. 

Several studies agree about more adverse effects after the second dose, such as fatigue, nausea/vomiting, headache, or arthralgia/myalgia [21,23,29,43]. Nonetheless, all of them are considered minor adverse effects.

Adverse obstetrical outcomes were not described in association with COVID-19 vaccines [51]. The abortion rate was similar to that of the general population [51]. 

On the other hand, the presence of mARN vaccines in some milk samples (maximum 2 ng/mL) [41] were reported, which does not damage the newborn [41]. 

### 3.2. Efficacy 

A recently published study among 487 pregnant women with SARS-CoV-2 infection showed that vaccinated patients had an 80% lower risk for developing pneumonia and hospital admission due to COVID-19 than unvaccinated patients. Moreover, vaccinated pregnant patients with COVID-19 were associated to a lower composite adverse maternal outcome and requirement of antibiotics, corticosteroids, and oxygen therapy compared to unvaccinated patients [26]. Furthermore, no severe COVID-19 was found among pregnant patients vaccinated with at least two doses [26]. Theiler et al. studied a cohort of 2002 pregnant women in which the unvaccinated patients underwent a higher incidence of COVID-19 compared with the vaccinated patients [23]. 

### 3.3. Immunogenicity

Most of the studies focus their aim on the humoral response, quantifying the immunoglobulin isotypes. Several authors have studied immunoglobulin A (IgA). An Italian study analyzes the immunity of breastfed babies by mothers vaccinated during lactation [47]. Only the IgA1 isotype was found in milk, and the study was unable to demonstrate significant mucosal IgA2. The authors did not find a significant amount of antibodies in babies’ buccal swabs or feces [47]. Thus, they conclude that vaccination induces a strong Immunoglobulin G (IgG) humoral response in maternal serum and is lower in breast milk (10–150 times fewer immunoglobulins in milk than in maternal serum) [47].

Golan et al. did not find IgA in milk in 25% of the mothers. Additionally, 83% of them had children older than 5.5 months. The authors conclude that there is less IgA in the mother’s milk of older babies [34]. Another study found no relationship between IgA and the age of the newborn [35].

If vaccination and natural infection are compared, the presence and dynamics of antibodies are different. A study that included 2312 women concluded that IgA was detectable in milk 10 months after the infection [39]. In a lactating vaccinated population, the authors describe a biphasic IgA response. It rises a week after the first dose and a week after the second dose, reaching 85% positivity, but decreases quickly [39]. Perl et al. showed that 86.1% of the samples were positive for IgA a week after the second dose, this value reducing to 65.7% of samples a week later [46]. Scrimin et al. showed the absence of IgA in serum and breast milk 20 days after the second vaccine dose [50]. However, a Brazilian study observed IgA rises from 2 weeks post-vaccination and peaks at 5–6 weeks [27]. Nonetheless, only 20 patients were included in this study [27]. Young et al. showed IgA increases in human milk only after the first dose, reducing after the second dose [17]. These data were confirmed by other studies [36]. 

Some studies also analyze the dynamics of IgG. Scrimin and Esteve-Palau et al. showed long-lasting IgG in both serum and breast milk after the second vaccine. IgG was detected four weeks [32,50] and even six months after vaccination, gradually decreasing its efficacy [45]. Perl et al. detected IgG antibodies in 97% of samples 5–6 weeks after the first dose [46]. Jakusszko et al. described that the IgG response was strongest seven days after the second vaccine dose [38]. Gray et al. also showed an increase in IgG in serum and milk after the second dose [36], and a Portuguese study concluded that the main response to vaccination was IgG-mediated [30]. 

A prospective cohort of 86 breastfeeding women, vaccinated or not, described similar IgG quantification in patients who were infected and recovered from COVID-19 disease after the first vaccine dose compared to patients vaccinated after the second dose without suffering the infection [25]. 

Regarding the relationship between milk and serum antibody levels, Golan et al. observed a positive relation measured 4–10 weeks after the second dose. However, levels were similar after the first dose [34]. Friedman et al. described that antibody response is rapid and highly synchronized between breastmilk and serum, reaching stabilization 14 days after the second dose [33]. Moreover, in 84% of the cases, IgG was detected in serum longer than in breast milk [24]. An Italian cohort did not find a correlation between serum and milk [37]. 

The ratio IgG-IgA has also been quantified [25,41]. Low et al. described that the amount of IgA and IgG reached its maximum at 3–7 days after the second dose. However, IgG is more stable and is even detected 4–6 weeks after vaccination [41]. 

Apart from A and G, other isotypes of immunoglobulin have been studied. Previous studies agree with Golan et al. who concluded that high levels of immunoglobulin M (IgM) and IgG are found in the serum of vaccinated mothers. IgG is multiplied by 6 with the second vaccine dose (which doesn’t occur with IgM) [34]. The function of IgG was not modified after pasteurization but it inactivated isotypes M and A [45].

In a Spanish cohort of 86 patients, 32 were vaccinated with AstraZeneca. Authors describe that the presence and persistence of specific antibodies against SARS-CoV-2 in breast milk depended on the type of vaccine, with this being stronger for the mRNA vaccines than the AstraZeneca one [25]. 

A more efficient passive immunity has also been described in vaccinated women (starting 16 days after the first dose) [48] than in women who recovered from COVID-19 infection. This finding was also described by Olearo et al., who compared infected lactating women who were vaccinated and not vaccinated. The transfer of antibodies to breast milk was significantly higher in women who recovered from COVID-19 and were vaccinated during lactation versus recovered unvaccinated women [44]. Even though the efficacy of passive immunity for the COVID-19 vaccine has been proven, it is weaker than passive immunity induced by other classical vaccines, such as the flu and whooping cough [49]. 

Atyeo et al. analyzed a cohort of 131 women to compare their response to vaccination. They observed that the titre of antibodies was similar. Nonetheless, the junction to the Fc receptor and the function of the antibody was induced later after the first vaccine in pregnant and lactating populations, compared to not pregnant and not lactating individuals [28]. This catches up after the second dose [28]. 

There is a stronger transfer of antibodies to the newborn if early vaccination occurs during pregnancy. A latency of weeks was described for vaccine response [42]. This latency was also observed for milk donors [42] as well as infected unvaccinated patients [26]. 

## 4. Discussion

In this systematic review of vaccination during pregnancy and breastfeeding, it was found that vaccination is safe for pregnant and breastfeeding women. The main adverse effects were pain at the injection site and fatigue. These women are not more susceptible to adverse effects than the general population [19]. Adverse effects, such as fatigue, headache, and myalgia, are more frequent after the second dose and are slightly more frequent after the Moderna vaccine [21,23,29,43].

Furthermore, the vaccine does not lead to adverse obstetrics outcomes [40,51]. Evidence refutes the alarm raised by preliminary studies, which warned of an 82% risk of spontaneous abortion [52]. 

The studies about the vaccine’s efficacy agree that vaccination in pregnant women reduces the risk of severe infection in this population [23,26], who are more susceptible to developing severe COVID-19 disease [5,6,9,11]. Vaccination also reduces the risk of developing pneumonia, hospital admission, and the requirement for antibiotics, corticosteroids, and oxygen therapy [26].

The humoral response to COVID-19 vaccination has been widely studied [17,25,30,32,33,34,36,38,39,41,46,47,50]. Recent studies focus on clarifying the mother–child transmission of the cellular response [31,35]. Following mRNA vaccination, immune transfer into breast milk occurs through a combination of spike-reactive secretory antibodies (SIgA) secreted by mammary mucosa-associated lymphoid tissue (MALT) (90% of the total Ig), IgG, T cells, and bioactive factors such as lactoferrin, oligosaccharides, and cytokines [50]. These lines of defense could create synergies by conferring both immediate (SIgA) and long-lasting (T cell) immunity [35]. T-lymphocytes survive and seed in the newborn’s respiratory and gastrointestinal tracts. Memory T cells are long-lived. Therefore, the protection transferred by milk can be present in the baby even after stopping breastfeeding [35]. In addition, a longer-lived memory B cell response has been observed in lactating women [35]. Therefore, breastmilk straddles an immediate antibody-mediated and long-lived cellular-mediated immune protection [31,35].

According to Pietrasanta et al., vaccination induces a stronger immune response in maternal serum than in milk because IgA was not found in infants’ mucosae [47]. This finding has a physiological reason, as IgA2 is the only one resistant to protease. When authors conclude that the immune response in milk is weaker, they could be underestimating natural passive immunity because the cellular response is not analyzed [47]. Regarding the evolution of IgA and the age of the newborn, there are contradictory conclusions: from none [35] to a negative relationship [34].

The vast majority of studies are consistent about immunoglobulin dynamics in response to vaccination and infection [25,30,32,33,34,38,41,45,46,50]. The presence and dynamics of antibodies are different. IgA levels was detectable in women’s serum seven days post vaccination. They decrease two weeks after vaccination, being undetectable twenty days after vaccination [39,46,50]. However, IgG could be detected four weeks after vaccination [32,46,50]. Therefore, vaccination is associated with a larger and more stable IgG response (IgA decreases faster than IgG), while infection is associated with a rapid and long-lasting IgA response [17,25,30,36]. The vaccination response is optimal using mRNA vaccines [25]. IgG is more stable [25,41] and is the only isotype not inactivated by pasteurization [45].

A Spanish study concluded that artificial active immunogenicity acquired after vaccination is similar to natural active immunogenicity developed after infection [25]. However, a more efficient passive immunity in vaccinated women [48] than in women who recovered from COVID-19 infection has also been described. Furthermore, passive immunity for the COVID-19 vaccine is weaker than passive immunity induced by other classical vaccines such as the flu and whooping cough [49]. 

A similar quantitative but lower efficacy immune response to the vaccine in pregnant and breastfeeding women has been described compared to the general population [28]. The immune response is equalized after the second dose [28]. Thus, pregnant and lactating women should be encouraged to complete the vaccination schedule. Moreover, it is optimal to do it as soon as possible to compensate for the immune response latency [28,42]. 

Most studies found a positive relation between milk and serum antibody levels [33,34,50], with the exception of the Italian cohort [37]. These contradictory data could be explained because the latter study only included ten patients in the cohort [37].

The findings of this review are consistent with the results of existing systematic reviews on this topic [53,54]. However, these reviews were limited by their inclusion of fewer studies written before publication. COVID-19 has posed a challenge to researchers and clinicians, who have had to update daily in the face of the significant growth of scientific literature at an unprecedented speed.

Several weaknesses in the underlying literature were identified. The sample size of most of the studies was not a randomized sample from the general population. The Healthy volunteer bias could also have been committed. Furthermore, there were many healthcare workers, as they were the first young population to be vaccinated. However, working in healthcare was not associated with an increased vaccine acceptance [13,14,15,16]. Interestingly, white and Asian pregnant individuals were more likely to accept vaccination [13]. 

The strengths of this study include a comprehensive search strategy and the inclusion of a large and updated number of studies regarding COVID-19 vaccination during pregnancy and breastfeeding. However, the heterogeneity of the data reported in the literature prevented the authors from performing a meta-analysis. Future studies are needed to investigate COVID-19 vaccines and their long-term consequences. Additionally, research concerning immunogenicity with a larger number of patients is required. 

## 5. Conclusions

This systematic review has shown the safety of COVID-19 vaccination during pregnancy and breastfeeding. The minor side effects were pain at the injection site and fatigue. This study also proves the efficacy of vaccination, given that it reduces the risk of severe COVID-19 in pregnant women.

Passive immunity, both in terms of cellular and humoral immune response, for the COVID-19 vaccine has been proven. The vaccination response is optimal using mRNA vaccines. Vaccination is associated with a larger and more stable IgG response and infection with a rapid and long-lasting IgA response.

Thus, COVID-19 vaccination is not only suggested but strongly recommended for pregnant and breastfeeding populations to protect mothers and newborns.

## Data Availability

Not applicable.

## Abbreviations

Messenger ribonucleic acid	mARN
Immunoglobulin A, G, M	IgA, IgG, IgM
